# What should I say? Testing ways to reduce fear and increase disclosure of incivility in reference checks

**DOI:** 10.1371/journal.pone.0290011

**Published:** 2023-08-15

**Authors:** Benjamin M. Walsh, Brittany Heighton, Chloe Dingens

**Affiliations:** Department of Management, Seidman College of Business, Grand Valley State University, Grand Rapids, MI, United States of America; Guangxi Normal University, CHINA

## Abstract

We utilize signaling theory as a foundation for testing ways to decrease reference providers’ fear of adverse consequences and increase disclosure of workplace incivility in reference checks. We focus on three reminders–commonly recommended by practitioners–that may be sent to reference providers in the instructions prior to the reference check: reminders of applicant consent, qualified privilege, and confidentiality. 420 supervisors were recruited via Prolific.co to complete a hypothetical reference check for the employee with whom they least like to work. Participants were randomly assigned to one of eight conditions in a two (applicant consent reminder: yes/no) X two (qualified privilege reminder: yes/no) X two (confidentiality reminder: yes/no) between-subjects design. Instructions before the reference check were manipulated in a manner that corresponded to their experimental condition, after which they completed measures of fear and incivility. Results showed no main effects, but two interactions. Applicant consent and qualified privilege interacted in relation to fear of adverse legal consequences, and confidentially and qualified privilege interacted in relation to reports of applicant incivility (*p* < .10). Collectively, our largely null findings suggest that reference checks may be a limited tool for incivility prevention.

## Introduction

*Workplace incivility* includes the “rude, condescending, and ostracizing acts that violate workplace norms of mutual respect” [1 p. 299]. Importantly, meta-analysis shows that workplace incivility is detrimental to targets and the organizations in which it occurs, as it is associated with lower job satisfaction and well-being, and increased turnover intent [2]. Incivility is also a precursor to other forms of mistreatment, including aggression [3], and employers may face negligent hiring lawsuits if their employees act aggressively towards customers [4, 5]. Thus, it is in the hiring organization’s best interest to avoid hiring people who are likely to be rude, uncivil, and aggressive at work.

Researchers and practitioners encourage organizations to use *reference checks* to help determine who has a history of mistreating others [4, 6–8], because such information can be used to determine who they should not hire. However, reference providers tend to avoid divulging negative information about applicants [9, 10]. Although failure to disclose negative information may stem from myriad factors (e.g., social exchange between applicant and reference provider, societal cultural values and practices), it is often suggested that reference providers may fear adverse legal (e.g., a defamation lawsuit brought on by the applicant), and interpersonal consequences (e.g., conflict with the applicant) [4, 5, 11], which may help explain the hesitation among reference providers to discuss unfavorable information. Thus, although reference checks are used by more than 90% of employers [12], and they are predictive of job performance [13, 14], their utility for identifying uncivil job applicants prior to being hired is uncertain. That is, unless we can identify ways to assuage these concerns and encourage reference providers to honestly disclose information about the extent to which job applicants are uncivil.

We utilize signaling theory [15] as a theoretical foundation for experimentally testing ways to decrease reference providers’ fear of adverse consequences and increase their disclosure of rude behaviors during reference checks. We focus on three signals that may be sent by hiring organizations and/or reference checking vendors to reference providers in the instructions provided prior to the reference check. Importantly, these are not our own suggestions; each is recommended by researchers and practitioners as a feature of sound reference-checking practice. They include reminders of: (a) *applicant consent* (i.e., the job applicant consented to reference checking) [16], (b) *qualified privilege* (i.e., that reference providers are immune from legal liability for disclosing factual work-related information) [5], and (c) *confidentiality* (i.e., the job applicant will not see the information provided in the reference check) [17].

Our study contributes to the literature in three ways. First, we answer recent calls from researchers to study reference checks as a tool for incivility prevention [18]. This is important because workplace incivility is a type of workplace mistreatment that requires “special attention to eradicate” [2 p. 15]. Researchers have long suggested that reference checks may be one means to help prevent workplace incivility [6], but research designed to test their effectiveness in that regard is very limited [18], so our research helps to fill this important gap.

Second, although researchers and practitioners recommend each of the reminders that we study to improve the utility of reference checks, we are unaware of research that has subjected them to empirical study, with the exception being confidentiality [17]. By utilizing an experimental methodology, and studying the reminders together rather than in isolation, we are able to draw stronger conclusion about their collective role in improving the value of the information gathered from reference checks. Our work in this regard helps to move the field in the direction of evidence-based practice [19], in which practitioners utilize methods that are supported via empirical research as opposed to mere belief or intuition.

Third, although researchers have studied fear of adverse consequences in performance management contexts [20, 21], we were unable to identify a scale of fear of adverse consequences specific to the context of pre-employment reference checks. We develop a set of items and provide initial evidence of their reliability and factor structure that employee selection and reference checking researchers can use in future research. We continue with a brief overview of the theoretical rationale for our hypothesized relationships.

### Signaling theory and hypotheses

Signaling theory suggests that people look for observable signals to help make decisions in uncertain situations [15]. Reference providers are faced with uncertainty because they need to determine what kinds of information they should disclose during pre-employment reference checks. This uncertainty is arguably greatest when reference providers are asked to complete a reference check for an employee for whom they have an unfavorable perception, such as someone who tends to be rude at work. Given the dilemma about what information to disclose in a reference check, we reasoned that reference providers would be motivated to search for signals to reduce their uncertainty. The most efficacious signals are those that are clearly observable [15]. Hiring organizations are in a good position to send such clear signals to reference providers in the instructions provided just prior to the completion of a reference check [18]. It is possible that such instructions may attenuate their fear of adverse consequences, and increase their disclosure of applicant workplace incivility.

Three signals are the focus of our research. First, hiring organizations are encouraged by practitioners to solicit a written consent from job applicants as an authorization to conduct reference checks [4, 22]. With consent in hand, hiring organizations can then communicate to reference providers that the applicant has consented and agreed to the reference check. Doing so may send a reassuring signal to reference providers that the applicant is aware of their participation in the reference check, which may assuage their fear of adverse consequences, and increase their reporting of any incivility engaged in by the applicant. Without such a signal of applicant consent, reference providers may be left wondering whether it is even appropriate to comply with the reference check, which may leave them fearful and unwilling to disclose deleterious information about the applicant to the hiring organization. Based on this rationale, we developed the following hypothesis.

*Hypothesis 1*: Reference providers who are reminded of applicant consent will report less fear of adverse consequences (H1a) and more workplace incivility (H1b) than reference providers who receive no reminder of applicant consent.

Second, hiring organizations are encouraged to remind reference providers of their qualified privilege in completing the reference check [5]. Many states and territories, though not all, have statutes that effectively protect reference providers from legal liability [23], so long as they honestly disclose factual information about job applicants in reference checks [4, 5]. Cooper asserts that such laws are expressly designed to reduce fear on the part of reference providers: “The goal of these statutes is to encourage employers to provide references, based on the expectation that qualified immunity in reference-based lawsuits will reduce employers’ fears of being sued” [22 p. 6]. Indeed, signaling to reference providers that they have qualified privilege may reduce their fear of adverse consequences, and encourage more honest disclosure of applicant workplace incivility. Without the reminder of qualified privilege, reference providers may remain fearful and unwilling to disclose negative information such as the applicant’s incivility. Reference providers are likely unaware that such legal protection even exists when a reminder is not provided in the instructions prior to the reference check [22]. Accordingly, we hypothesized:

*Hypothesis 2*: Reference providers who are reminded of qualified privilege will report less fear of adverse consequences (H2a) and more workplace incivility (H2b) than reference providers who receive no reminder of qualified privilege.

Third, hiring organizations/reference checking vendors are encouraged to ensure that the reference check is confidential, such that the job applicant is unable to view the content of the completed reference check that is offered by a specific reference provider [17]. For example, SkillSurvey works to ensure the confidentiality of their reference checking process [24]. Ceci and Peters showed that in reference checks submitted for graduate school, students tended to receive more negative evaluations in confidential reference checks when contrasted with nonconfidential reference checks [17]. Consequently, sending clear signals to reference providers that the reference check is confidential may reduce their fear of any adverse consequences, and increase reports of applicant workplace incivility. Without a reminder of confidentiality, reference providers may be more fearful and hesitant to reveal the applicant’s uncivil workplace behavior, as they may anticipate that the applicant will be able to view the content of the reference check.

*Hypothesis 3*: Reference providers who are reminded of confidentiality will report less fear of adverse consequences (H3a) and more workplace incivility (H3b) than reference providers who receive no reminder of confidentiality.

Finally, it is also possible for multiple signals to be sent and interpreted, which suggests the possibility for an interaction among signals, where one signal changes the impact of another [15]. We test the aforementioned three signals, which represents an opportunity for us to study potential interactions in relation to fear of adverse consequences and reports of applicant workplace incivility.

The most reassuring scenario for a reference provider may exist when two or even all three signals are observed prior to completing the reference check, which may lead reference providers to experience the lowest levels of fear of adverse consequences and report the highest levels of applicant workplace incivility. It may also be the case that the presence of one signal, such as the reminder of confidentiality, may change how other signals are interpreted, which may manifest in a differential relation to the outcomes we study. For instance, the reminder of applicant consent may function differently in relation to fear of adverse consequences and reports of applicant workplace incivility when the reference check is not confidential versus situations where confidentiality is assured. Accordingly, we also explored as a research question the extent to which interactions exist among the three signals that we studied.

*Research Question*: Do reminders of applicant consent, qualified privilege, and confidentiality interact (e.g., two- or three-way interactions) to explain variance in fear of adverse consequences and applicant workplace incivility?

## Materials and methods

### Overview

Prior to beginning our study, we secured approval to carry out our research through the Grand Valley State University institutional review board (Protocol 21-248-H, written consent obtained via online survey). Data and materials for our research are available via OSF at https://osf.io/rtczg/. We collected data on August 10–11 in 2021 from people in supervisory positions located in the United States, Canada, or United Kingdom who were asked to complete a hypothetical, web-based (via Qualtrics) pre-employment reference check as part of their participation in our study. Informed consent was obtained from all participants. Our use of a hypothetical reference check was necessary given our need to manipulate aspects of the reference check, while negating ethical dilemmas of doing so in a real reference checking context. Our approach is also consistent with methods used in the reference checking literature [11, 18]. Moreover, our focus was on supervisors because they are commonly asked to complete pre-employment reference checks [11]. Participants were recruited via Prolific (www.prolific.co). Prolific takes several steps to ensure that participants are legitimate [25], and Walter and colleagues [26] showed that data collected from such sources are comparable to samples collected from organizations. We also considered guidance by Aguinis and colleagues [27] and included several checks to help ensure the integrity of the data (e.g., CAPTCHA verification, insufficient effort responding items).

Participants were asked to complete the reference check for “the employee you least like to work with in your primary job.” We chose these instructions for several reasons. First, although applicants are asked to list preferred sources for reference checks, employers often ask for references from individuals not provided by the applicant, what the Society for Human Resource Management (SHRM) refers to as backdoor references [28]. We suspect that in a real reference checking context, such backdoor references would likely include individuals, such as previous supervisors, who would be more likely to disclose areas of concern about the applicant’s behavior (e.g., incivility). Thus, we believe that our instructions enhance the external validity of our findings, especially to contexts in which such backdoor references are gathered.

Second, we wanted to minimize demand characteristics insofar as we did not want to directly inform participants that our research interest (in part) was in their perceptions of an employee’s incivility, which we believed would occur if we had directly focused participant’s attention on an uncivil employee. We nonetheless reasoned that uncivil behavior would influence who participants decided to reflect upon; in addition to perceptions of competence, perceptions of warmth–to which uncivil behavior directly relate [29]–reflect fundamental aspects of social perception [30]. Comparisons to a pilot sample in which different instructions were used support this conclusion. In particular, independent samples *t*-tests comparing our study sample (*N* = 420) with a pilot sample (*N* = 138) collected via Prolific on June 29, 2021 in which participants were asked about “the employee with whom you interact most frequently” showed that the instructions used in our study led to higher fear of legal consequences (*M* = 1.85, *SD* = 1.02 vs. *M* = 1.52, *SD* = .87), fear of interpersonal consequences (*M* = 2.34, *SD* = 1.20 vs. *M* = 1.59, *SD* = .86) and reports of applicant workplace incivility (*M* = 2.83, *SD* = 1.14 vs. *M* = 1.85, *SD* = .89) than in the pilot data (all *p*s < .05). These data suggest that employee incivility played a key role in determining who participants reflected upon while completing the reference check, and attest to the external validity of our study in inducing moderate levels of fear, despite entailing a hypothetical reference check that was completed by participants.

### Participants

Responses were received from 436 individuals in supervisory positions. Prior to data analysis, we screened for insufficient effort responding (IER) [31] in a two-step process. First, in the survey were two instructed response items (e.g., “Please select ‘less often than others’”) [31]. We allowed participants to fail one item (skip the item or provide an incorrect response), which could result from “transient measurement error” [31 p. 297]. One participant was removed for failing both items. Second, we included a self-report IER item towards the end of the survey: “I have paid no attention to this survey so far”. We retained only participants who responded with “strongly disagree” or “disagree”, which resulted in the removal of an additional 15 participants (16 participants removed for IER in total, 3.7% of the sample). The remaining 420 participants were primarily female (82.1%), with 88.8% identifying as white. We were notified by Prolific after data collection that our study was affected by a surge in female participant signups, which led to high representation of female participants in our study. Participants worked 38.6 hours/week on average (*SD* = 11.0), and had known the applicant about whom they completed the reference check for 2.7 years on average (*SD* = 2.6). Participants identified 61.9% of the applicants as female, and they were mostly white (72.6%) and Hispanic (9.5%).

### Procedure

Participants were asked to reflect upon the employee they least like to work with in their primary job, after which they were presented with the following instructions, which were inspired by instructions used by König and colleagues [9]:

Imagine that the employee you’ve been asked to think about had to leave their job for private reasons.

Your former employee–now “the applicant”–found an interesting job at a new employer, and they mentioned your name as a previous supervisor.

Now imagine that you have been asked to complete a pre-employment reference check for the applicant.

After these instructions, participants began the reference check with three questions informed by Taylor and colleagues’ [14] reference check that were not the focus of our hypotheses: (a) “How long (in years) have you known the applicant? (response open-ended), (b) “What is the nature of your work relationship with the applicant? I am a _____ of the applicant.” (we focused only on participants who selected “1” [*manager/supervisor*]), and (c) “What is your personal relationship with the applicant?” (response options = “1” [*none*], “2” [*acquaintances*], “3” [*friends*], “4” [*very good friends*]. Then participants were randomly assigned to one of 8 conditions in a 2 (applicant consent reminder: yes/no) X 2 (qualified privilege reminder: yes/no) X 2 (confidentiality reminder: yes/no) between-subjects design, so one condition had no reminders, one condition had all three reminders, three conditions had two reminders, and three conditions had one reminder. Exact instructions are included at https://osf.io/rtczg/.

### Measures

The following focal measures were completed by participants after the aforementioned instructions, and they were presented in random order. Additional questions commonly found in reference checks were included to enhance the realism of the reference check (e.g., “Would you rehire the applicant?”), although they were not the focus of our research questions. Table 1 shows reliabilities for all measures. Higher scores reflect higher levels of all constructs.

**Table 1:** Zero order correlations and reliability estimates.

Variable	*M*	*SD*	1	2	3	4	5	6
1. Applicant Incivility	2.83	1.14	(.94)					
2. Fear (L)	1.85	1.02	.34**	(.95)				
3. Fear (I)	2.34	1.20	.51**	.64**	(.93)			
4. Applicant Consent	.50	.50	-.03	.03	.05	--		
5. Qualified Privilege	.52	.50	.05	-.04	-.05	.01	--	
6. Confidentiality	.49	.50	-.06	.02	-.06	-.05	.03	--

*N* = 420. The manipulations for the reminders of applicant consent, qualified privilege, and confidentiality were scored “0” (*no reminder provided*) and “1” (*yes reminder provided*). Fear (L) = fear of adverse legal consequences. Fear (I) = fear of adverse interpersonal consequences. Cronbach alpha internal consistency reliability estimates are presented along the diagonal, where applicable.

* *p* < .05.

** *p* < .01.

#### Fear of adverse consequences

Although researchers have studied fear of adverse consequences in performance management contexts [20, 21], we were unable to identify a scale specific to reference checking. Thus, six items were generated to measure fear of adverse consequences when completing a reference check, consistent with discussions in the literature [5, 22]. Instructions were “Recall the instructions you were presented on the previous page. While imagining that you have been asked to complete a pre-employment reference check for the applicant, please evaluate the extent to which you would be…”

Items measuring fear of legal consequences included: “Worried about being sued for defamation of character by the applicant because of the reference check”, “Concerned that the applicant might file a lawsuit against me because of the reference check”, and “Fearful of legal concerns with the applicant because of the reference check”. Items measuring fear of interpersonal consequences included: “Worried about getting into a disagreement with the applicant because of the reference check”, “Concerned about having an argument with the applicant because of the reference check,” and “Fearful about getting into a fight with the applicant because of the reference check”. Responses were on a “1” (*strongly disagree*) to “5” (*strongly agree*) scale. In a confirmatory factor analysis with maximum likelihood estimation, the two-factor model (χ^2^ [8] = 9.23, *p* = .323, CFI = 1.00, RMSEA = .02, SRMR = .02) fit the data better than a one-factor model (χ^2^ [9] = 679.88, *p* < .001, CFI = .73, RMSEA = .42, SRMR = .13) and corresponded to the two types of fear (i.e., fear of legal consequences and fear of interpersonal consequences).

#### Applicant workplace incivility

Blau and Andersson’s [32] measure was used to measure applicant incivility. Instructions were “Compared to others you have known in their position, how often has the applicant exhibited the following behaviors while at work?” An example item is “Put down others or were condescending to them in some way.” Responses were on the relative scale recommended by Walsh and colleagues [33] for measuring incivility in pre-employment reference checks, ranging from “1” (*much less often than others*) to “5” (*much more often than others*).

#### Manipulation checks

We used factual manipulation checks to assess their effectiveness [34]. Participants were asked three questions after these instructions: “Before completing the reference check, were you asked to imagine that…”. The questions were: (a) “the applicant signed a consent form authorizing you to complete the reference check (i.e., applicant consent)?”, (b) “you cannot be sued for providing factual, honest information in reference checks about the job performance of current or former employees (i.e., qualified privilege)?”, and (c) “the information you provide in the reference check is completely confidential (i.e., confidentiality).” Responses were “0” (*no*) or “1” (*yes*). The factual manipulation checks were included at the end of the reference check.

## Results

We first investigated the effectiveness of our manipulations via chi-square analyses (χ^2^). Results showed that each of our manipulations were effective, although there was variability in the percentage of correct responses across the factual manipulation checks. First, the manipulation of applicant consent was effective (χ^2^[1] = 109.16, *p* < .001), such that 92.4% of participants who received the reminder of applicant consent correctly said they did, and 54.8% who did not receive the consent reminder said they did not. Second, the qualified privilege manipulation functioned as expected (χ^2^[1] = 155.62, *p* < .001), as 88.1% of participants who were reminded of qualified privilege correctly responded that they received the reminder, and 71.8% of participants who were not reminded correctly noted that they did not receive the qualified privilege reminder. Finally, the manipulation of confidentiality was also effective (χ^2^[1] = 108.42, *p* < .001), with 97.1% of participants who received the confidentiality reminder correctly responding that they had received it, although only 47.2% of participants who had not received the reminder correctly responded that no confidentiality reminder was sent.

We proceeded to test our hypotheses using separate hierarchical ordinary least squares (OLS) regression analyses, in which each outcome variable (i.e., fear of legal consequences, fear of interpersonal consequences, and applicant workplace incivility) was regressed on the main effects and interaction terms. Main effects were entered in step 1, two-way interaction terms were entered in step 2, and the three-way interaction term was entered in step 3. We tested hypotheses using all data, regardless of whether an individual passed all manipulation checks. This approach aligns with what West and colleagues [35] refer to as an intent to treat analysis, which represents a conservative test of hypotheses. Likewise, this approach aligns with the practical reality of reference checks such that all data would be utilized in an actual pre-employment reference check setting, regardless of whether any signals sent to reference providers were actually received.

Table 2 displays results from the regression analyses. No support was observed for any main effects. Specifically, Hypothesis 1 was unsupported as the reminder of applicant consent did not influence fear of adverse legal (*b* = .07, *p* = .490) or interpersonal (*b* = .11, *p* = .337) consequences, and it did not increase reports of applicant workplace incivility (*b* = -.08, *p* = .485). Hypothesis 2 was also unsupported as the qualified privilege reminder failed to reduce fear of adverse legal (*b* = -.09, *p* = .385) or interpersonal consequences (*b* = -.13, *p* = .273), and did not promote increased reporting of applicant workplace incivility (*b* = .13, *p* = .259). Finally, Hypothesis 3 was unsupported because the confidentiality reminder also did not decrease fear of adverse legal (*b* = .05, *p* = .607) or interpersonal (*b* = -.13, *p* = .281) consequences, and failed to increase reports of applicant workplace incivility (*b* = -.14, *p* = .195).

**Table 2:** Results from ordinary least squares regression analysis.

		Dependent Variable														
		Fear of Legal Consequences					Fear of Interpersonal Consequences					Applicant Workplace Incivility				
Model	Variable	*b*	*s*.*e*.	*p*	Δ *R*^2^	Δ *R*	*b*	*s*.*e*.	*p*	Δ *R*^2^	Δ *R*	*b*	*s*.*e*.	*p*	Δ *R*^2^	Δ *R*
1	Intercept	1.84	.10	< .001	.003	.058	2.41	.12	< .001	.008	.091	2.87	.11	< .001	.008	.088
	AC (H1)	.07	.10	.490			.11	.12	.337			-.08	.11	.485		
	QP (H2)	-.09	.10	.385			-.13	.12	.273			.13	.11	.259		
	C (H3)	.05	.10	.607			-.13	.12	.281			-.14	.11	.195		
2	Intercept	1.79	.13	< .001	.019	.091	2.25	.16	< .001	.009	.039	2.88	.15	< .001	.013	.057
	AC	.32	.17	.061			.43	.20	.035			.09	.19	.642		
	QP	-.02	.17	.901			.03	.21	.883			.09	.19	.656		
	C	-.08	.17	.639			.03	.21	.904			-.34	.19	.078		
	AC*QP (RQ)	-.44	.20	.028			-.32	.24	.175			-.31	.22	.160		
	AC*C (RQ)	-.06	.20	.775			-.31	.24	.194			-.02	.22	.943		
	QP*C (RQ)	.32	.20	.111			.01	.24	.974			.40	.22	.072		
3	Intercept	1.76	.14	< .001	.001	.002	2.29	.17	< .001	.001	.004	2.98	.16	< .001	.006	.018
	AC	.37	.20	.061			.36	.23	.128			-.08	.22	.710		
	QP	.03	.20	.878			-.05	.24	.851			-.09	.23	.681		
	C	-.03	.20	.884			-.05	.24	.835			-.52	.22	.021		
	AC*QP	-.54	.28	.054			-.18	.33	.590			.03	.31	.931		
	AC*C	-.16	.29	.571			-.15	.34	.650			.34	.32	.281		
	QP*C	.22	.28	.445			.15	.33	.646			.75	.31	.018		
	AC*QP*C (RQ)	.20	.40	.609			-.29	.47	.537			-.69	.44	.119		
	Total *R*^2^	.023					.018					.027				
	Total *R*	.151					.134					.163				

*N* = 420. AC = Applicant consent reminder. QP = qualified privilege reminder. C = confidentiality reminder (all scored “0” [*no*], “1” [*yes*]). Coefficients were interpreted at the step at which they were first entered (e.g., main effects in model 1, two-way interactions in model 2). Coefficients corresponding to hypothesis tests (H1-H3) and the research question (RQ) are noted.

**Fig 1:**
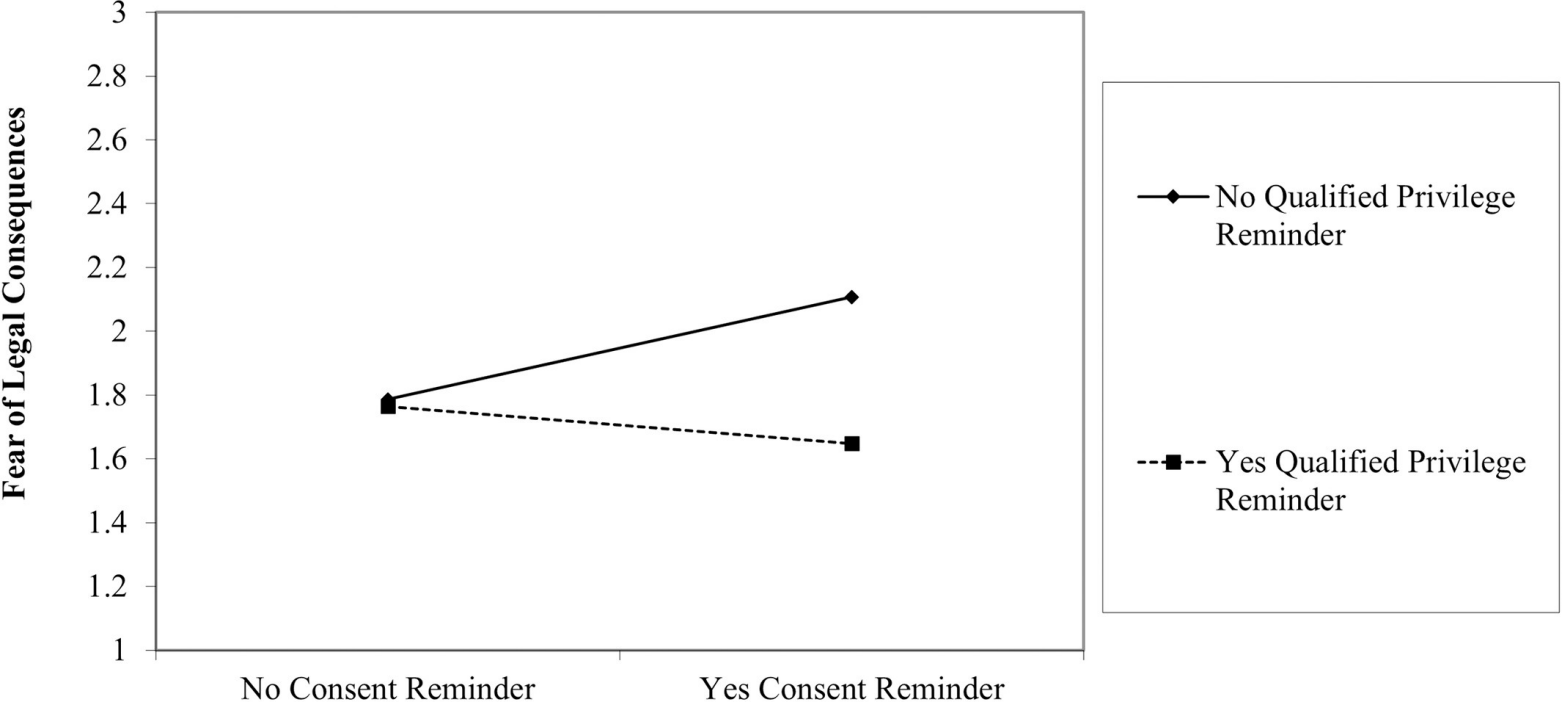
Interaction between applicant consent and qualified privilege reminders in relation to fear of adverse legal consequences.

**Fig 2:**
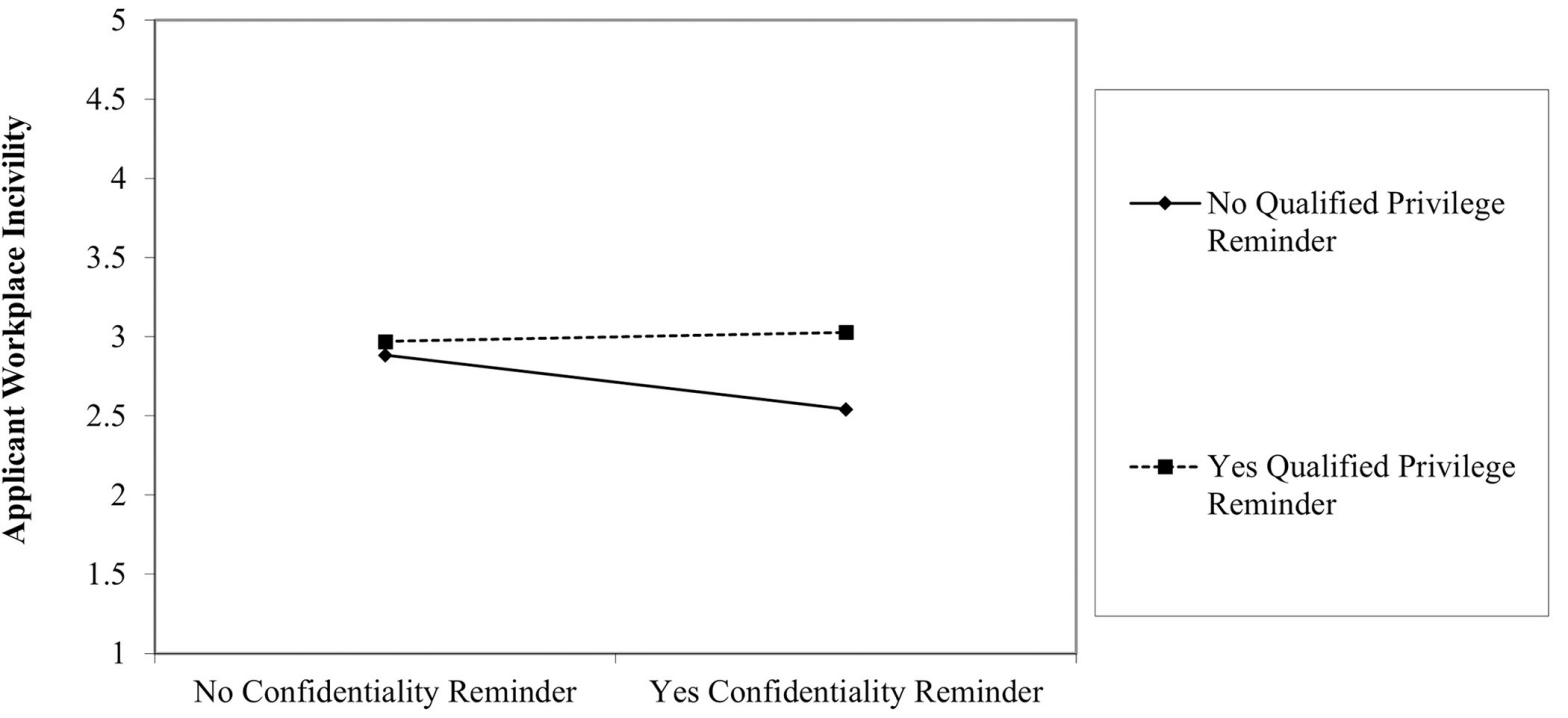
Interaction between confidentiality and qualified privilege reminders in relation to applicant workplace incivility.

We investigated interactions as part of our Research Question. Given the difficulty in observing interactions, we considered Aguinis’ [36] and McClelland and Judd’s [37] suggestions and accepted a higher Type I error rate for their detection (*p* < .10). We observed two interactions which we plotted with the help of Dawson’s [38] tools. First, applicant consent and qualified privilege interacted to explain variance in fear of adverse legal consequences (*b* = -.44, *p* = .028; Fig 1). Whether a reminder of qualified privilege was provided made no difference in fear without the presence of a reminder of applicant consent. With a consent reminder, fear was highest when no qualified privilege reminder was offered, and lowest with the reminder of qualified privilege. Second, confidentially and qualified privilege interacted in relation to applicant workplace incivility (*b* = .40, *p* = .072; Fig 2). Results showed that whether a qualified privilege reminder was provided mattered only when a confidentiality reminder was also provided. The highest reports of applicant incivility occurred when both confidentiality and qualified privilege reminders were sent. The lowest reports of applicant incivility occurred when a confidentiality reminder was provided, but no qualified privilege reminder was sent to reference providers.

## Discussion

We sought to apply signaling theory to experimentally test whether three signals (reminders) sent to reference providers could decrease their fear and increase their reporting of applicant workplace incivility. Below we summarize the theoretical implications and limitations of our study, as well as the practical implications of our research for hiring organizations.

### Implications for theory, research, and practice

Our study offers implications for the literature on reference checks. First, it is important to note that the signals and their interactions explained only a small amount of variability in reference provider’s fear of adverse consequences and reports of applicant workplace incivility. We found no evidence for direct effects of the signals we studied. Thus, although researchers and practitioners encourage hiring organizations to employ the practices we studied and remind reference providers if the practices are in effect, findings from our study suggests that they have a limited impact on fear of adverse consequences and reports of applicant workplace incivility when used in isolation. This observation is pertinent as we seek to move toward evidence-based practice [19], specifically with respect to the use of pre-employment reference checks, and especially in terms of screening for workplace incivility among applicants.

With that said, we did observe some evidence for interactions among the signals that were sent to reference providers, each of which explained a small proportion of the variability in the outcomes that we studied. In both of the observed interactions, the reminder of qualified privilege appeared to slightly modify the impact of another reminder: the effect of confidentiality for reports of applicant incivility, and the effect of applicant consent for fear of adverse legal consequences. For reports of applicant workplace incivility, it is intriguing to note that a reminder of confidentiality alone, without qualified privilege, led to the lowest reports of incivility. This suggests that confidentiality alone is not entirely reassuring for reference providers, a finding which stands in contrast with prior research [17]. For fear of adverse legal consequences, the highest level of fear was observed when participants were reminded of applicant consent, but there was no accompanying qualified privilege reminder. In this scenario, participants know that they have been authorized by the applicant to complete the reference check but they do not have the legal protection that qualified privilege offers, thus leading to the experience of fear. Collectively, these results attest to the significance of ensuring qualified privilege for reference providers [22], and suggest that additional research is needed to elucidate its impact on the disclosure of information in pre-employment reference checks.

Our research also offers implications for signaling theory. Specifically, our research provides additional evidence that signals can interact to influence receiver (e.g., reference provider) behavior [15]. This is consistent with the findings of Walsh et al. [18]. Walsh et al. [18] observed that prospective reference providers reflect on the different behaviors of their colleagues (e.g., in-role performance, incivility) as providers decide whether to recommend their colleague for employment, or what they referred to as willingness to recommend. Walsh et al. [18] also observed evidence for interactions among signals, such that perceptions of colleague in-role performance and incivility interacted to explain variability in willingness to recommend. Our research reinforces the need to study multiple signals at once, rather than study the impact of any one signal in isolation.

We also contribute to research on potential ways to improve the effectiveness of reference checks. With the exception of confidentiality [17], we were unaware of research testing whether the reminders of applicant consent and qualified privilege are effective at improving the utility of reference checks. This is despite the fact that researchers encourage hiring organizations to remind providers of qualified privilege [5], and to solicit consent from applicants to conduct reference checks [16]. We provide additional evidence that hiring organizations/reference checking vendors have the ability to send signals to reference providers, that these signals will be received by many (if not all) reference providers (as noted in our tests of manipulation checks), and that the signals can influence (however slightly) the behavior of reference providers.

We encourage additional research into ways to improve the utility of reference checks, and we envision several possibilities to that end. For instance, in our control conditions, no reminders of applicant consent, confidentiality, or qualified privilege were sent. In future studies, researchers may find value in studying the implications for fear and reports of incivility of signaling to reference providers that they do *not* have qualified privilege, that the reference check is *not* confidential, and/or that the applicant did *not* consent to reference checking. We envision such scenarios as being somewhat unrealistic in practice, especially for some of the reminders that we studied. For example, carrying out reference checks for job applicants who refused to consent to the reference checking process could expose the hiring organization to legal liability. This explains why we utilized our particular methodology, as we reasoned that it was more likely for no reminder to be sent in actual reference checking contexts. Nonetheless, this represents one possibility for future investigation, and a way to potentially strengthen the manipulations that we utilized in the present research.

We believe that several practical implications extend from our study. Reference checks are among the most widely used selection methods [12], and they can offer valuable information for hiring organizations [13]. We encourage organizations to continue utilizing reference checks, albeit with some modifications. First, if organizations have significant concerns about workplace incivility, then we encourage organizations to solicit information about applicant workplace incivility via reference checks, just as other incivility researchers have encouraged [6–8]. However, the manner in which this information is gathered is critical. We concur with Walsh et al. [33] that such information about applicant incivility should be gathered using a standardized and structured format, similar to the approach we used in our research, rather than inquiring generally about areas where the job applicant needs to improve [24]. Reference providers should be asked to report the extent to which the applicant engaged in various uncivil behaviors at work using a standardized scale such as Blau and Andersson’s [32] measure which we utilized, with responses captured on a relative (versus absolute) scale to help minimize lenience in ratings [33]. We believe that this approach–specifically inquiring about incivility in a standardized and structured format–may enable hiring organizations to uncover more incivility among job applicants when contrasted with alternative approaches, such as asking references about applicant’s areas for improvement. This is because reference providers often avoid delineating specific areas of improvement when prompted using an open-ended question format [24].

Second, fear of adverse legal consequences was lowest and reports of applicant incivility were highest when providers were reminded of qualified privilege and confidentiality (for incivility) or applicant consent (for fear). Thus, we encourage hiring organizations and reference checking vendors to include all three reminders in instructions prior to gathering data from reference checks, assuming qualified privilege applies in their locale [23]. Indeed, the inclusion of such reminders is a relatively simple intervention that our research suggests may help to increase the value of the information derived from pre-employment reference checks.

Finally, given the small effects that we observed, and our limited ability to influence reference provider fear and reports of incivility via the manipulations we studied, we ultimately encourage hiring organizations to use *multiple* methods to screen for and help prevent workplace incivility in organizations. Reference checks are but one, limited tool for incivility prevention, and organizations concerned about preventing incivility should employ initiatives at all stages of the employee lifecycle [8].

### Limitations and future research directions

Our study has several limitations that should be considered by readers as they interpret our findings. First, our study involved a hypothetical reference check. We believe this approach was necessary for the ethical reasons we described earlier; namely, that manipulating the reference checking process for actual job applicants could influence their ability to secure employment. In addition, our instructions did induce a moderate level of fear of adverse legal and interpersonal consequences and reports of applicant workplace incivility compared to a pilot sample. However, future research may attempt to study these variables in an actual high-stakes selection context, if such a possibility became available. Researchers may find that the effects we observed may become stronger if studied in a high-stakes context wherein a job applicant’s future employment depends on the input of the reference provider.

Second, although we investigated the impacts of three reminders that may be sent to reference providers on their reports of applicant workplace incivility, our study is limited insofar as we did not account for any additional explanations for the uncivil behavior of the applicant, as perceived by the prospective reference provider. Research suggests that people engage in instigated incivility as a result of various situational and personal explanations [39], the latter of which may be especially pertinent to employee selection and reference checking contexts. Future research on the use of reference checks for incivility prevention could attempt to account for these additional person-based precursors of instigated workplace incivility.

Third, because our data were collected via an online survey, all participants essentially completed a web-based reference check. Web-based reference checking is commonly used [13], but other methods are used as well (e.g., telephone, letters). Given our method, we did not explore potential differences in the effects of applicant consent, confidentiality, and qualified privilege across different reference checking methods. Future research should consider this possibility. The method by which references are collected may influence compliance rates by reference providers [11], so we suspect that it is also possible that the reference checking method may influence the effects that were tested in the present study.

Fourth, our analysis of the effectiveness of our manipulation checks suggested that participants who were sent the reminders of applicant consent, confidentiality, and qualified privilege tended to receive the reminders, which attests to their observability as signals [15]. However, individuals who were not sent the reminders at times responded that they had, incorrectly, received the reminders. These errors were especially true for participants who did not receive the reminder of applicant consent and confidentiality. We suspect that these errors may have stemmed from participants conflating the words “consent” and “confidentiality” that were included in our informed consent for their research participation with our factual manipulation checks that also asked them whether they were reminded of consent and confidentiality.

Fifth, the composition of our sample also represents a potential limitation of our research. In particular, our sample was comprised primarily of white female supervisors. We acknowledge the possibility that our results may not generalize to more diverse samples. Future research should seek to gather data from more demographically diverse groups in order to investigate the generalizability of our findings.

Sixth, our data were cross-sectional. We believe that this approach is justifiable as it represents the way reference checks are actually conducted, such that providers respond at a single time, and it is consistent with prior research on reference checks [11, 18]. In addition, our predictors were all experimentally manipulated, and the effects we observed involved two-way interactions among the manipulated variables. Siemsen et al. [40] showed that common method bias is unlikely to account for such interaction effects. However, future research could take a longitudinal approach to explore potential causal relations among constructs.

One such possibility is a potential causal relation between reference provider’s fear of adverse consequences and their reports of applicant workplace incivility, because the direction of that relation is unclear. Interestingly, fear of adverse legal and interpersonal consequences and applicant incivility as reported by reference providers were positively correlated in our study. Research shows that fearful individuals attempt to minimize risk in their decision making [41]. This finding suggests that people who are more fearful would report less (not more) applicant workplace incivility in an attempt to minimize the risk of any adverse legal or interpersonal consequences. In contrast, it is also possible that fear is an outcome of reports of applicant workplace incivility, such that reference providers experience fear when asked to reflect upon and report about an uncivil job applicant when completing the reference check. Our cross-sectional data provide limited information on this causal relation. The use of longitudinal research methods may provide clearer insight into the true nature of the relation between fear of adverse legal and interpersonal consequences and reports of applicant workplace incivility.

## Supporting information

S1 Checklist STROBE statement—checklist of items that should be included in reports of observational studies. (DOCX)
