# Supplementary material for: What should I say? Testing ways to reduce fear and increase disclosure of incivility in reference checks
Source: PLoS One. 2023 Aug 15;18(8):e0290011. doi: 10.1371/journal.pone.0290011 (PMC10426956; doi:10.1371/journal.pone.0290011)
Supplement: S1 Checklist — (DOCX) [file pone.0290011.s001.docx]

STROBE Statement—checklist of items that should be included in reports of observational studies

|  | Item No. | Recommendation | Page  No. | Relevant text from manuscript |
| --- | --- | --- | --- | --- |
| **Title and abstract** | 1 | (*a*) Indicate the study’s design with a commonly used term in the title or the abstract | 2 | Participants were randomly assigned to one of eight conditions in a two (applicant consent reminder: yes/no) X two (qualified privilege reminder: yes/no) X two (confidentiality reminder: yes/no) between-subjects design |
|  |  | (*b*) Provide in the abstract an informative and balanced summary of what was done and what was found | 2 | Participants were randomly assigned to one of eight conditions in a two (applicant consent reminder: yes/no) X two (qualified privilege reminder: yes/no) X two (confidentiality reminder: yes/no) between-subjects design. Instructions before the reference check were manipulated in a manner that corresponded to their experimental condition, after which they completed measures of fear and incivility. Results showed no main effects, but two interactions. Applicant consent and qualified privilege interacted in relation to fear of adverse legal consequences, and confidentially and qualified privilege interacted in relation to reports of applicant incivility (*p* < .10). Collectively, our largely null findings suggest that reference checks may be a limited tool for incivility prevention. |
| Introduction | | | |  |
| Background/rationale | 2 | Explain the scientific background and rationale for the investigation being reported | 3-5 | *Workplace incivility* includes the “rude, condescending, and ostracizing acts that violate workplace norms of mutual respect” (Cortina et al., 2017, p. 299). Importantly, meta-analysis shows that workplace incivility is detrimental to targets and the organizations in which it occurs, as it is associated with lower job satisfaction and well-being, and increased turnover intent (Yao et al., 2022). Incivility is also a precursor to­­ other forms of mistreatment, including aggression (Taylor & Kluemper, 20­­12), and employers may face negligent hiring lawsuits if their employees act aggressively towards customers (Kondrasuk et al., 2001; Levashina & Campion, 2009). Thus, it is in the hiring organization’s best interest to avoid hiring people who are likely to be rude, uncivil, and aggressive at work.  Researchers and practitioners encourage organizations to use *reference checks* to help determine who has a history of mistreating others (Andersson & Pearson, 1999; Kondrasuk et al., 2001; Lim et al., 2008; Porath & Pearson, 2013), because such information can be used to determine who they should not hire. However, reference providers tend to avoid divulging negative information about applicants (König et al., 2017; Nicklin & Roch, 2009). Although failure to disclose negative information may stem from myriad factors (e.g., social exchange between applicant and reference provider, societal cultural values and practices), it is often suggested that reference providers may fear adverse legal (e.g., a defamation lawsuit brought on by the applicant), and interpersonal consequences (e.g., conflict with the applicant; Hedricks et al., 2019; Kondrasuk et al., 2001; Levashina & Campion, 2009), which may help explain the hesitation among reference providers to discuss unfavorable information. Thus, although reference checks are used by more than 90% of employers (Heneman et al., 2015), and they are predictive of job performance (Hedricks et al., 2013; Taylor et al., 2004), their utility for identifying uncivil job applicants prior to being hired is uncertain. That is, unless we can identify ways to assuage these concerns and encourage reference providers to honestly disclose information about the extent to which job applicants are uncivil.  We utilize signaling theory (Connelly et al., 2011) as a theoretical foundation for experimentally testing ways to decrease reference providers’ fear of adverse consequences and increase their disclosure of rude behaviors during reference checks. We focus on three signals that may be sent by hiring organizations and/or reference checking vendors to reference providers in the instructions provided prior to the reference check. Importantly, these are not our own suggestions; each is recommended by researchers and practitioners as a feature of sound reference-checking practice. They include reminders of: (a) *applicant consent* (i.e., the job applicant consented to reference checking; Edwards & Kleiner, 2002), (b) *qualified privilege* (i.e., that reference providers are immune from legal liability for disclosing factual work-related information; Levashina & Campion, 2009), and (c) *confidentiality* (i.e., the job applicant will not see the information provided in the reference check; Ceci & Peters, 1984).  Our study contributes to the literature in three ways. First, we answer recent calls from researchers to study reference checks as a tool for incivility prevention (Walsh et al., 2021). This is important because workplace incivility is a type of workplace mistreatment that Yao et al. (2022) emphasize requires “special attention to eradicate” (p. 15). Researchers have long suggested that reference checks may be one means to help prevent workplace incivility (e.g., Andersson & Pearson, 1999), but research designed to test their effectiveness in that regard is very limited (cf., Walsh et al., 2021), so our research helps to fill this important gap.  Second, although researchers and practitioners recommend each of the reminders that we study to improve the utility of reference checks, we are unaware of research that has subjected them to empirical study, with the exception being confidentiality (e.g., Ceci & Peters, 1984). By utilizing an experimental methodology, and studying the reminders together rather than in isolation, we are in a position to draw stronger conclusion about their collective role in improving the value of the information gathered from reference checks. Our work in this regard helps to move the field in the direction of evidence-based practice (Barends et al., 2014), in which practitioners utilize methods that are supported via empirical research as opposed to mere belief or intuition.  Third, although researchers have studied fear of adverse consequences in performance management contexts (e.g., Canali, 2011; Spence & Keeping, 2010; Waung & Highhouse, 1997), we were unable to identify a scale of fear of adverse consequences specific to the context of pre-employment reference checks. We develop a set of items and provide initial evidence of their reliability and factor structure that employee selection and reference checking researchers can use in future research. We continue with a brief overview of the theoretical rationale for our hypothesized relationships. |
| Objectives | 3 | State specific objectives, including any prespecified hypotheses | 6-8 | *Hypothesis 1:* Reference providers who are reminded of applicant consent will report less fear of adverse consequences (H1a) and more workplace incivility (H1b) than reference providers who receive no reminder of applicant consent.  *Hypothesis 2:* Reference providers who are reminded of qualified privilege will report less fear of adverse consequences (H2a) and more workplace incivility (H2b) than reference providers who receive no reminder of qualified privilege.  *Hypothesis 3:* Reference providers who are reminded of confidentiality will report less fear of adverse consequences (H3a) and more workplace incivility (H3b) than reference providers who receive no reminder of confidentiality.  *Research Question:* Do reminders of applicant consent, qualified privilege, and confidentiality interact (e.g., two- or three-way interactions) to explain variance in fear of adverse consequences and applicant workplace incivility? |
| Methods | | | |  |
| Study design | 4 | Present key elements of study design early in the paper | 8-11 | We collected data in 2021 from people in supervisory positions located in the United States, Canada, or United Kingdom who were asked to complete a hypothetical, web-based (via Qualtrics) pre-employment reference check as part of their participation in our study. Informed consent was obtained from all participants. Our use of a hypothetical reference check was necessary given our need to manipulate aspects of the reference check, while negating ethical dilemmas of doing so in a real reference checking context. Our approach is also consistent with methods used in the reference checking literature (e.g., Hedricks et al., 2019; Walsh et al., 2021). Moreover, our focus was on supervisors because they are commonly asked to complete pre-employment reference checks (Hedricks et al., 2019). Participants were recruited via Prolific (www.prolific.co). Prolific takes a number of steps to ensure that participants are legitimate (Bradley, 2018), and Walter et al. (2019) showed that data collected from such sources are comparable to samples collected from organizations. We also considered guidance by Aguinis et al. (2021) and included several checks to help ensure the integrity of the data (e.g., CAPTCHA verification, insufficient effort responding items).  Participants were asked to complete the reference check for “the employee you least like to work with in your primary job.” We chose these instructions for several reasons. First, although applicants are asked to list preferred sources for reference checks, employers often ask for references from individuals not provided by the applicant, what the Society for Human Resource Management (SHRM) refers to as “backdoor references” (Grensing-Pophal, 2019). We suspect that in a real reference checking context, such backdoor references would likely include individuals, such as previous supervisors, who would be more likely to disclose areas of concern about the applicant’s behavior (e.g., incivility). Thus, we believe that our instructions enhance the external validity of our findings, especially to contexts in which such backdoor references are gathered.  Second, we wanted to minimize demand characteristics insofar as we did not want to directly inform participants that our research interest (in part) was in their perceptions of an employee’s incivility, which we believed would occur if we had directly focused participant’s attention on an uncivil employee. We nonetheless reasoned that uncivil behavior would influence who participants decided to reflect upon; in addition to perceptions of competence, perceptions of warmth – to which uncivil behavior directly relate (Porath et al., 2015) – reflect fundamental aspects of social perception (Cuddy et al., 2008). Comparisons to a pilot sample in which different instructions were used support this conclusion. In particular, independent samples *t*-tests comparing our study sample (*N* = 420) with a pilot sample (*N* = 138) in which participants were asked about “the employee with whom you interact most frequently” showed that the instructions used in our study led to higher fear of legal consequences (*M* = 1.85, *SD* = 1.02 vs. *M* = 1.52, *SD* = .87), fear of interpersonal consequences (*M* = 2.34, *SD* = 1.20 vs. *M* = 1.59, *SD* = .86) and reports of applicant workplace incivility (*M* = 2.83, *SD* = 1.14 vs. *M* = 1.85, *SD* = .89) than in the pilot data (all *p*s < .05). These data suggest that employee incivility played a key role in determining who participants reflected upon while completing the reference check, and attest to the external validity of our study in inducing moderate levels of fear, despite entailing a hypothetical reference check that was completed by participants.  **Participants**  Responses were received from 436 individuals in supervisory positions. Prior to data analysis, we screened for insufficient effort responding (IER; McGonagle et al., 2016) in a two-step process. First, in the survey were two instructed response items (e.g., “Please select ‘less often than others’”; McGonagle et al., 2016). We allowed participants to fail one item (skip the item or provide an incorrect response), which could result from “transient measurement error” (McGonagle et al., 2016, p. 297). One participant was removed for failing both items. Second, we included a self-report IER item towards the end of the survey: “I have paid no attention to this survey so far”. We retained only participants who responded with “strongly disagree” or “disagree”, which resulted in the removal of an additional 15 participants (16 participants removed for IER in total, 3.7% of the sample). The remaining 420 participants were primarily female (82.1%)^^[[1]](#footnote-1)^^, with 88.8% identifying as white. Participants worked 38.6 hours/week on average (*SD* = 11.0), and had known the applicant about whom they completed the reference check for 2.7 years on average (*SD* = 2.6). Participants identified 61.9% of the applicants as female, and they were mostly white (72.6%) and Hispanic (9.5%).  **Procedure**  Participants were asked to reflect upon the employee they least like to work with in their primary job, after which they were presented with the following instructions, which were inspired by instructions used by König et al. (2017):  Imagine that the employee you’ve been asked to think about had to leave their job for private reasons.  Your former employee – now “the applicant” – found an interesting job at a new employer, and they mentioned your name as a previous supervisor.  Now imagine that you have been asked to complete a pre-employment reference check for the applicant.  After these instructions, participants began the reference check with three questions informed by Taylor et al.’s (2004) reference check that were not the focus of our hypotheses: (a) “How long (in years) have you known the applicant? (response open-ended), (b) “What is the nature of your work relationship with the applicant? I am a _____ of the applicant.” (we focused only on participants who selected “1” [*manager/supervisor*]), and (c) “What is your personal relationship with the applicant?” (response options = “1” [*none*], “2” [*acquaintances*], “3” [*friends*], “4” [*very good friends*]. Then participants were randomly assigned to one of 8 conditions in a 2 (applicant consent reminder: yes/no) X 2 (qualified privilege reminder: yes/no) X 2 (confidentiality reminder: yes/no) between-subjects design, so one condition had no reminders, one condition had all three reminders, three conditions had two reminders, and three conditions had one reminder. Exact instructions are included at the [OSF site](https://osf.io/rtczg/?view_only=7fe90112402e46e1b5b61d6935c0b556). |
| Setting | 5 | Describe the setting, locations, and relevant dates, including periods of recruitment, exposure, follow-up, and data collection | 8 | Prior to beginning our study, we secured approval to carry out our research through the institutional review board at our institution. Data and materials for our research are available via the [Open Science Framework](https://osf.io/rtczg/?view_only=7fe90112402e46e1b5b61d6935c0b556) (OSF) at https://osf.io/rtczg/?view_only=7fe90112402e46e1b5b61d6935c0b556. We collected data on August 10-11 in 2021 from people in supervisory positions located in the United States, Canada, or United Kingdom who were asked to complete a hypothetical, web-based (via Qualtrics) pre-employment reference check as part of their participation in our study. |
| Participants | 6 | (*a*) *Cohort study*—Give the eligibility criteria, and the sources and methods of selection of participants. Describe methods of follow-up  *Case-control study*—Give the eligibility criteria, and the sources and methods of case ascertainment and control selection. Give the rationale for the choice of cases and controls  *Cross-sectional study*—Give the eligibility criteria, and the sources and methods of selection of participants | 8 | We collected data on August 10-11 in 2021 from people in supervisory positions located in the United States, Canada, or United Kingdom who were asked to complete a hypothetical, web-based (via Qualtrics) pre-employment reference check as part of their participation in our study. |
|  |  | (*b*) *Cohort study*—For matched studies, give matching criteria and number of exposed and unexposed  *Case-control study*—For matched studies, give matching criteria and the number of controls per case |  |  |
| Variables | 7 | Clearly define all outcomes, exposures, predictors, potential confounders, and effect modifiers. Give diagnostic criteria, if applicable | 11-13 | **Measures**  The following focal measures were completed by participants after the aforementioned instructions, and they were presented in random order. Additional questions commonly found in reference checks were included to enhance the realism of the reference check (e.g., “Would you rehire the applicant?”), although they were not the focus of our research questions. Table 1 shows reliabilities for all measures. Higher scores reflect higher levels of all constructs.  ***Fear of Adverse Consequences***  Although researchers have studied fear of adverse consequences in performance management contexts (e.g., Canali, 2011; Spence & Keeping, 2010; Waung & Highhouse, 1997), we were unable to identify a scale specific to reference checking. Thus, six items were generated to measure fear of adverse consequences when completing a reference check, consistent with discussions in the literature (e.g., Cooper, 2001; Levashina & Campion, 2009). Instructions were “Recall the instructions you were presented on the previous page. While imagining that you have been asked to complete a pre-employment reference check for the applicant, please evaluate the extent to which you would be…”  Items measuring fear of legal consequences included: “Worried about being sued for defamation of character by the applicant because of the reference check”, “Concerned that the applicant might file a lawsuit against me because of the reference check”, and “Fearful of legal concerns with the applicant because of the reference check”. Items measuring fear of interpersonal consequences included: “Worried about getting into a disagreement with the applicant because of the reference check”, “Concerned about having an argument with the applicant because of the reference check,” and “Fearful about getting into a fight with the applicant because of the reference check”. Responses were on a “1” (*strongly disagree*) to “5” (*strongly agree*) scale. In a confirmatory factor analysis with maximum likelihood estimation, the two-factor model (χ^2^ [8] = 9.23, *p* = .323, CFI = 1.00, RMSEA = .02, SRMR = .02) fit the data better than a one-factor model (χ^2^ [9] = 679.88, *p* < .001, CFI = .73, RMSEA = .42, SRMR = .13) and corresponded to the two types of fear (i.e., fear of legal consequences and fear of interpersonal consequences).  ***Applicant Workplace Incivility***  Blau and Andersson’s (2005) measure was used to measure applicant incivility. Instructions were “Compared to others you have known in their position, how often has the applicant exhibited the following behaviors while at work?” An example item is “Put down others or were condescending to them in some way.” Responses were on the relative scale recommended by Walsh et al. (2019) for measuring incivility in pre-employment reference checks, ranging from “1” (*much less often than others*) to “5” (*much more often than others*).  ***Manipulation Checks***  We used factual manipulation checks to assess their effectiveness (Kane & Barabas, 2019). Participants were asked three questions after these instructions: “Before completing the reference check, were you asked to imagine that…”. The questions were: (a) “the applicant signed a consent form authorizing you to complete the reference check (i.e., applicant consent)?”, (b) “you cannot be sued for providing factual, honest information in reference checks about the job performance of current or former employees (i.e., qualified privilege)?”, and (c) “the information you provide in the reference check is completely confidential (i.e., confidentiality).” Responses were “0” (*no*) or “1” (*yes*). The factual manipulation checks were included at the end of the reference check. |
| Data sources/ measurement | 8* | For each variable of interest, give sources of data and details of methods of assessment (measurement). Describe comparability of assessment methods if there is more than one group | 11-13 | **Measures**  The following focal measures were completed by participants after the aforementioned instructions, and they were presented in random order. Additional questions commonly found in reference checks were included to enhance the realism of the reference check (e.g., “Would you rehire the applicant?”), although they were not the focus of our research questions. Table 1 shows reliabilities for all measures. Higher scores reflect higher levels of all constructs.  ***Fear of Adverse Consequences***  Although researchers have studied fear of adverse consequences in performance management contexts (e.g., Canali, 2011; Spence & Keeping, 2010; Waung & Highhouse, 1997), we were unable to identify a scale specific to reference checking. Thus, six items were generated to measure fear of adverse consequences when completing a reference check, consistent with discussions in the literature (e.g., Cooper, 2001; Levashina & Campion, 2009). Instructions were “Recall the instructions you were presented on the previous page. While imagining that you have been asked to complete a pre-employment reference check for the applicant, please evaluate the extent to which you would be…”  Items measuring fear of legal consequences included: “Worried about being sued for defamation of character by the applicant because of the reference check”, “Concerned that the applicant might file a lawsuit against me because of the reference check”, and “Fearful of legal concerns with the applicant because of the reference check”. Items measuring fear of interpersonal consequences included: “Worried about getting into a disagreement with the applicant because of the reference check”, “Concerned about having an argument with the applicant because of the reference check,” and “Fearful about getting into a fight with the applicant because of the reference check”. Responses were on a “1” (*strongly disagree*) to “5” (*strongly agree*) scale. In a confirmatory factor analysis with maximum likelihood estimation, the two-factor model (χ^2^ [8] = 9.23, *p* = .323, CFI = 1.00, RMSEA = .02, SRMR = .02) fit the data better than a one-factor model (χ^2^ [9] = 679.88, *p* < .001, CFI = .73, RMSEA = .42, SRMR = .13) and corresponded to the two types of fear (i.e., fear of legal consequences and fear of interpersonal consequences).  ***Applicant Workplace Incivility***  Blau and Andersson’s (2005) measure was used to measure applicant incivility. Instructions were “Compared to others you have known in their position, how often has the applicant exhibited the following behaviors while at work?” An example item is “Put down others or were condescending to them in some way.” Responses were on the relative scale recommended by Walsh et al. (2019) for measuring incivility in pre-employment reference checks, ranging from “1” (*much less often than others*) to “5” (*much more often than others*).  ***Manipulation Checks***  We used factual manipulation checks to assess their effectiveness (Kane & Barabas, 2019). Participants were asked three questions after these instructions: “Before completing the reference check, were you asked to imagine that…”. The questions were: (a) “the applicant signed a consent form authorizing you to complete the reference check (i.e., applicant consent)?”, (b) “you cannot be sued for providing factual, honest information in reference checks about the job performance of current or former employees (i.e., qualified privilege)?”, and (c) “the information you provide in the reference check is completely confidential (i.e., confidentiality).” Responses were “0” (*no*) or “1” (*yes*). The factual manipulation checks were included at the end of the reference check. |
| Bias | 9 | Describe any efforts to address potential sources of bias | 9 | Participants were recruited via Prolific (www.prolific.co). Prolific takes a number of steps to ensure that participants are legitimate (Bradley, 2018), and Walter et al. (2019) showed that data collected from such sources are comparable to samples collected from organizations. We also considered guidance by Aguinis et al. (2021) and included several checks to help ensure the integrity of the data (e.g., CAPTCHA verification, insufficient effort responding items). |
| Study size | 10 | Explain how the study size was arrived at | N/A | This was determined mainly by our limited budget. |

Continued on next page

| Quantitative variables | 11 | Explain how quantitative variables were handled in the analyses. If applicable, describe which groupings were chosen and why | 13-14 | We first investigated the effectiveness of our manipulations via chi-square analyses (χ^2^). Results showed that each of our manipulations were effective, although there was variability in the percentage of correct responses across the factual manipulation checks. First, the manipulation of applicant consent was effective (χ^2^[1] = 109.16, *p* < .001), such that 92.4% of participants who received the reminder of applicant consent correctly said they did, and 54.8% who did not receive the consent reminder said they did not. Second, the qualified privilege manipulation functioned as expected (χ^2^[1] = 155.62, *p* < .001), as 88.1% of participants who were reminded of qualified privilege correctly responded that they received the reminder, and 71.8% of participants who were not reminded correctly noted that they did not receive the qualified privilege reminder. Finally, the manipulation of confidentiality was also effective (χ^2^[1] = 108.42, *p* < .001), with 97.1% of participants who received the confidentiality reminder correctly responding that they had received it, although only 47.2% of participants who had not received the reminder correctly responded that no confidentiality reminder was sent.    We proceeded to test our hypotheses using hierarchical ordinary least squares (OLS) regression. Main effects were entered in step 1, two-way interaction terms were entered in step 2, and the three-way interaction term was entered in step 3. We tested hypotheses using all data, regardless of whether an individual passed all manipulation checks. This approach aligns with what West et al. (2000) refer to as an intent to treat analysis, which represents a conservative test of hypotheses. Likewise, this approach aligns with the practical reality of reference checks such that all data would be utilized in an actual pre-employment reference check setting, regardless of whether any signals sent to reference providers were actually received. |
| --- | --- | --- | --- | --- |
| Statistical methods | 12 | (*a*) Describe all statistical methods, including those used to control for confounding | 13-14 | We first investigated the effectiveness of our manipulations via chi-square analyses (χ^2^). Results showed that each of our manipulations were effective, although there was variability in the percentage of correct responses across the factual manipulation checks. First, the manipulation of applicant consent was effective (χ^2^[1] = 109.16, *p* < .001), such that 92.4% of participants who received the reminder of applicant consent correctly said they did, and 54.8% who did not receive the consent reminder said they did not. Second, the qualified privilege manipulation functioned as expected (χ^2^[1] = 155.62, *p* < .001), as 88.1% of participants who were reminded of qualified privilege correctly responded that they received the reminder, and 71.8% of participants who were not reminded correctly noted that they did not receive the qualified privilege reminder. Finally, the manipulation of confidentiality was also effective (χ^2^[1] = 108.42, *p* < .001), with 97.1% of participants who received the confidentiality reminder correctly responding that they had received it, although only 47.2% of participants who had not received the reminder correctly responded that no confidentiality reminder was sent.  We proceeded to test our hypotheses using hierarchical ordinary least squares (OLS) regression. Main effects were entered in step 1, two-way interaction terms were entered in step 2, and the three-way interaction term was entered in step 3. We tested hypotheses using all data, regardless of whether an individual passed all manipulation checks. This approach aligns with what West et al. (2000) refer to as an intent to treat analysis, which represents a conservative test of hypotheses. Likewise, this approach aligns with the practical reality of reference checks such that all data would be utilized in an actual pre-employment reference check setting, regardless of whether any signals sent to reference providers were actually received. |
|  |  | (*b*) Describe any methods used to examine subgroups and interactions | 13-14 | We first investigated the effectiveness of our manipulations via chi-square analyses (χ^2^). Results showed that each of our manipulations were effective, although there was variability in the percentage of correct responses across the factual manipulation checks. First, the manipulation of applicant consent was effective (χ^2^[1] = 109.16, *p* < .001), such that 92.4% of participants who received the reminder of applicant consent correctly said they did, and 54.8% who did not receive the consent reminder said they did not. Second, the qualified privilege manipulation functioned as expected (χ^2^[1] = 155.62, *p* < .001), as 88.1% of participants who were reminded of qualified privilege correctly responded that they received the reminder, and 71.8% of participants who were not reminded correctly noted that they did not receive the qualified privilege reminder. Finally, the manipulation of confidentiality was also effective (χ^2^[1] = 108.42, *p* < .001), with 97.1% of participants who received the confidentiality reminder correctly responding that they had received it, although only 47.2% of participants who had not received the reminder correctly responded that no confidentiality reminder was sent.  We proceeded to test our hypotheses using hierarchical ordinary least squares (OLS) regression. Main effects were entered in step 1, two-way interaction terms were entered in step 2, and the three-way interaction term was entered in step 3. We tested hypotheses using all data, regardless of whether an individual passed all manipulation checks. This approach aligns with what West et al. (2000) refer to as an intent to treat analysis, which represents a conservative test of hypotheses. Likewise, this approach aligns with the practical reality of reference checks such that all data would be utilized in an actual pre-employment reference check setting, regardless of whether any signals sent to reference providers were actually received. |
|  |  | (*c*) Explain how missing data were addressed | 10-11 | Responses were received from 436 individuals in supervisory positions. Prior to data analysis, we screened for insufficient effort responding (IER; McGonagle et al., 2016) in a two-step process. First, in the survey were two instructed response items (e.g., “Please select ‘less often than others’”; McGonagle et al., 2016). We allowed participants to fail one item (skip the item or provide an incorrect response), which could result from “transient measurement error” (McGonagle et al., 2016, p. 297). One participant was removed for failing both items. Second, we included a self-report IER item towards the end of the survey: “I have paid no attention to this survey so far”. We retained only participants who responded with “strongly disagree” or “disagree”, which resulted in the removal of an additional 15 participants (16 participants removed for IER in total, 3.7% of the sample). The remaining 420 participants were primarily female (82.1%)^^[[2]](#footnote-2)^^, with 88.8% identifying as white. Participants worked 38.6 hours/week on average (*SD* = 11.0), and had known the applicant about whom they completed the reference check for 2.7 years on average (*SD* = 2.6). Participants identified 61.9% of the applicants as female, and they were mostly white (72.6%) and Hispanic (9.5%). |
|  |  | (*d*) *Cohort study*—If applicable, explain how loss to follow-up was addressed  *Case-control study*—If applicable, explain how matching of cases and controls was addressed  *Cross-sectional study*—If applicable, describe analytical methods taking account of sampling strategy | N/A |  |
|  |  | (*e*) Describe any sensitivity analyses | N/A |  |
| Results | | | | |
| Participants | 13* | (a) Report numbers of individuals at each stage of study—eg numbers potentially eligible, examined for eligibility, confirmed eligible, included in the study, completing follow-up, and analysed | 10-11 | Responses were received from 436 individuals in supervisory positions. Prior to data analysis, we screened for insufficient effort responding (IER; McGonagle et al., 2016) in a two-step process. First, in the survey were two instructed response items (e.g., “Please select ‘less often than others’”; McGonagle et al., 2016). We allowed participants to fail one item (skip the item or provide an incorrect response), which could result from “transient measurement error” (McGonagle et al., 2016, p. 297). One participant was removed for failing both items. Second, we included a self-report IER item towards the end of the survey: “I have paid no attention to this survey so far”. We retained only participants who responded with “strongly disagree” or “disagree”, which resulted in the removal of an additional 15 participants (16 participants removed for IER in total, 3.7% of the sample). The remaining 420 participants were primarily female (82.1%)^^[[3]](#footnote-3)^^, with 88.8% identifying as white. Participants worked 38.6 hours/week on average (*SD* = 11.0), and had known the applicant about whom they completed the reference check for 2.7 years on average (*SD* = 2.6). Participants identified 61.9% of the applicants as female, and they were mostly white (72.6%) and Hispanic (9.5%). |
|  |  | (b) Give reasons for non-participation at each stage | N/A |  |
|  |  | (c) Consider use of a flow diagram | N/A |  |
| Descriptive data | 14* | (a) Give characteristics of study participants (eg demographic, clinical, social) and information on exposures and potential confounders | 10-11 | The remaining 420 participants were primarily female (82.1%)^^[[4]](#footnote-4)^^, with 88.8% identifying as white. Participants worked 38.6 hours/week on average (*SD* = 11.0), and had known the applicant about whom they completed the reference check for 2.7 years on average (*SD* = 2.6). Participants identified 61.9% of the applicants as female, and they were mostly white (72.6%) and Hispanic (9.5%). |
|  |  | (b) Indicate number of participants with missing data for each variable of interest | N/A |  |
|  |  | (c) *Cohort study*—Summarise follow-up time (eg, average and total amount) | N/A |  |
| Outcome data | 15* | *Cohort study*—Report numbers of outcome events or summary measures over time | N/A |  |
|  |  | *Case-control study—*Report numbers in each exposure category, or summary measures of exposure | N/A |  |
|  |  | *Cross-sectional study—*Report numbers of outcome events or summary measures | 29 | Table 1 |
| Main results | 16 | (*a*) Give unadjusted estimates and, if applicable, confounder-adjusted estimates and their precision (eg, 95% confidence interval). Make clear which confounders were adjusted for and why they were included | 30 | Table 2 |
|  |  | (*b*) Report category boundaries when continuous variables were categorized | N/A |  |
|  |  | (*c*) If relevant, consider translating estimates of relative risk into absolute risk for a meaningful time period | N/A |  |

Continued on next page

| Other analyses | 17 | Report other analyses done—eg analyses of subgroups and interactions, and sensitivity analyses | 15 | We investigated interactions as part of our Research Question. Given the difficulty in observing interactions, we considered Aguinis’ (1995) and McClelland and Judd’s (1993) suggestions and accepted a higher Type I error rate for their detection (*p* < .10). We observed two interactions which we plotted with the help of Dawson’s (2014) tools. First, applicant consent and qualified privilege interacted to explain variance in fear of adverse legal consequences (*b* = -.44, *p* = .028; Figure 1). Whether a reminder of qualified privilege was provided made no difference in fear without the presence of a reminder of applicant consent. With a consent reminder, fear was highest when no qualified privilege reminder was offered, and lowest with the reminder of qualified privilege. Second, confidentially and qualified privilege interacted in relation to applicant workplace incivility (*b* = .40, *p* = .072; Figure 2). Results showed that whether a qualified privilege reminder was provided mattered only when a confidentiality reminder was also provided. The highest reports of applicant incivility occurred when both confidentiality and qualified privilege reminders were sent. The lowest reports of applicant incivility occurred when a confidentiality reminder was provided, but no qualified privilege reminder was sent to reference providers. |
| --- | --- | --- | --- | --- |
| Discussion | | | | |
| Key results | 18 | Summarise key results with reference to study objectives | 16-18 | Our study offers implications for the literature on reference checks. First, it is important to note that the signals and their interactions explained only a small amount of variability in reference provider’s fear of adverse consequences and reports of applicant workplace incivility. We found no evidence for direct effects of the signals we studied. Thus, although researchers and practitioners encourage hiring organizations to employ the practices we studied and remind reference providers if the practices are in effect, findings from our study suggests that they have a limited impact on fear of adverse consequences and reports of applicant workplace incivility when used in isolation. This observation is pertinent as we seek to move toward evidence-based practice (Barends et al., 2014), specifically with respect to the use of pre-employment reference checks, and especially in terms of screening for workplace incivility among applicants.  With that said, we did observe some evidence for interactions among the signals that were sent to reference providers, each of which explained a small proportion of the variability in the outcomes that we studied. In both of the observed interactions, the reminder of qualified privilege appeared to slightly modify the impact of another reminder: the effect of confidentiality for reports of applicant incivility, and the effect of applicant consent for fear of adverse legal consequences. For reports of applicant workplace incivility, it is intriguing to note that a reminder of confidentiality alone, without qualified privilege, led to the lowest reports of incivility. This suggests that confidentiality alone is not entirely reassuring for reference providers, a finding which stands in contrast with prior research (Ceci & Peters, 1984). For fear of adverse legal consequences, the highest level of fear was observed when participants were reminded of applicant consent, but there was no accompanying qualified privilege reminder. In this scenario, participants know that they have been authorized by the applicant to complete the reference check but they do not have the legal protection that qualified privilege offers, thus leading to the experience of fear. Collectively, these results attest to the significance of ensuring qualified privilege for reference providers (Cooper, 2001), and suggest that additional research is needed to elucidate its impact on the disclosure of information in pre-employment reference checks.  Our research also offers implications for signaling theory. Specifically, our research provides additional evidence that signals can interact to influence receiver (e.g., reference provider) behavior (Connelly et al., 2011). This is consistent with the findings of Walsh et al. (2021). Walsh et al. observed that prospective reference providers reflect on the different behaviors of their colleagues (e.g., in-role performance, incivility) as providers decide whether to recommend their colleague for employment, or what they referred to as willingness to recommend. Walsh et al. also observed evidence for interactions among signals, such that perceptions of colleague in-role performance and incivility interacted to explain variability in willingness to recommend. Our research reinforces the need to study multiple signals at once, rather than study the impact of any one signal in isolation.  We also contribute to research on potential ways to improve the effectiveness of reference checks. With the exception of confidentiality (cf., Ceci & Peters, 1984), we were unaware of research testing whether the reminders of applicant consent and qualified privilege are effective at improving the utility of reference checks. This is despite the fact that researchers encourage hiring organizations to remind providers of qualified privilege (e.g., Levashina & Campion, 2009), and to solicit consent from applicants to conduct reference checks (e.g., Edwards & Kleiner, 2002). We provide additional evidence that hiring organizations/reference checking vendors have the ability to send signals to reference providers, that these signals will be received by many (if not all) reference providers (as noted in our tests of manipulation checks), and that the signals can influence (however slightly) the behavior of reference providers.  We encourage additional research into ways to improve the utility of reference checks, and we envision several possibilities to that end. For instance, in our control conditions, no reminders of applicant consent, confidentiality, or qualified privilege were sent. In future studies, researchers may find value in studying the implications for fear and reports of incivility of signaling to reference providers that they do *not* have qualified privilege, that the reference check is *not* confidential, and/or that the applicant did *not* consent to reference checking. We envision such scenarios as being somewhat unrealistic in practice, especially for some of the reminders that we studied. For example, carrying out reference checks for job applicants who refused to consent to the reference checking process could expose the hiring organization to legal liability. This explains why we utilized our particular methodology, as we reasoned that it was more likely for no reminder to be sent in actual reference checking contexts. Nonetheless, this represents one possibility for future investigation, and a way to potentially strengthen the manipulations that we utilized in the present research. |
| Limitations | 19 | Discuss limitations of the study, taking into account sources of potential bias or imprecision. Discuss both direction and magnitude of any potential bias | 18-20 | Our study has several limitations that should be considered by readers as they interpret our findings. First, our study involved a hypothetical reference check. We believe this approach was necessary for the ethical reasons we described earlier; namely, that manipulating the reference checking process for actual job applicants could influence their ability to secure employment. In addition, our instructions did induce a moderate level of fear of adverse legal and interpersonal consequences and reports of applicant workplace incivility compared to a pilot sample. However, future research may attempt to study these variables in an actual high-stakes selection context, if such a possibility became available. Researchers may find that the effects we observed may become stronger if studied in a high-stakes context wherein a job applicant’s future employment depends on the input of the reference provider.  Second, because our data were collected via an online survey, all participants essentially completed a web-based reference check. Web-based reference checking is commonly used (Hedricks et al., 2013), but other methods are used as well (e.g., telephone, letters). Given our method, we did not explore potential differences in the effects of applicant consent, confidentiality, and qualified privilege across different reference checking methods. Future research should consider this possibility. The method by which references are collected may influence compliance rates by reference providers (Hedricks et al., 2019), so we suspect that it is also possible that the reference checking method may influence the effects that were tested in the present study.  Third, our analysis of the effectiveness of our manipulation checks suggested that participants who were sent the reminders of applicant consent, confidentiality, and qualified privilege tended to receive the reminders, which attests to their observability as signals (Connelly et al., 2011). However, individuals who were not sent the reminders at times responded that they had, incorrectly, received the reminders. These errors were especially true for participants who did not receive the reminder of applicant consent and confidentiality. We suspect that these errors may have stemmed from participants conflating the words “consent” and “confidentiality” that were included in our informed consent for their research participation with our factual manipulation checks that also asked them whether they were reminded of consent and confidentiality.  Fourth, the composition of our sample also represents a potential limitation of our research. In particular, our sample was comprised primarily of white female supervisors. We acknowledge the possibility that our results may not generalize to more diverse samples. Future research should seek to gather data from more demographically diverse groups in order to investigate the generalizability of our findings.  Fifth, our data were cross-sectional. We believe that this approach is justifiable as it represents the way reference checks are actually conducted, such that providers respond at a single time, and it is consistent with prior research on reference checks (e.g., Hedricks et al., 2019; Walsh et al., 2021). In addition, our predictors were all experimentally manipulated, and the effects we observed involved two-way interactions among the manipulated variables. Siemsen et al. (2010) showed that common method bias is unlikely to account for such interaction effects. However, future research could take a longitudinal approach to explore potential causal relations among constructs. |
| Interpretation | 20 | Give a cautious overall interpretation of results considering objectives, limitations, multiplicity of analyses, results from similar studies, and other relevant evidence | 21-22 | Finally, given the small effects that we observed, and our limited ability to influence reference provider fear and reports of incivility via the manipulations we studied, we ultimately encourage hiring organizations to use *multiple* methods to screen for and help prevent workplace incivility in organizations. Reference checks are but one, limited tool for incivility prevention, and organizations concerned about preventing incivility should employ initiatives at all stages of the employee lifecycle (Porath & Pearson, 2013). |
| Generalisability | 21 | Discuss the generalisability (external validity) of the study results | 19 | Fourth, the composition of our sample also represents a potential limitation of our research. In particular, our sample was comprised primarily of white female supervisors. We acknowledge the possibility that our results may not generalize to more diverse samples. Future research should seek to gather data from more demographically diverse groups in order to investigate the generalizability of our findings. |
| Other information | |  | | |
| Funding | 22 | Give the source of funding and the role of the funders for the present study and, if applicable, for the original study on which the present article is based | N/A |  |

*Give information separately for cases and controls in case-control studies and, if applicable, for exposed and unexposed groups in cohort and cross-sectional studies.

**Note:** An Explanation and Elaboration article discusses each checklist item and gives methodological background and published examples of transparent reporting. The STROBE checklist is best used in conjunction with this article (freely available on the Web sites of PLoS Medicine at http://www.plosmedicine.org/, Annals of Internal Medicine at http://www.annals.org/, and Epidemiology at http://www.epidem.com/). Information on the STROBE Initiative is available at www.strobe-statement.org.

1. We were notified by Prolific after data collection that our study was affected by a surge in female participant signups, which led to high representation of female participants in our study. [↑](#footnote-ref-1)
2. We were notified by Prolific after data collection that our study was affected by a surge in female participant signups, which led to high representation of female participants in our study. [↑](#footnote-ref-2)
3. We were notified by Prolific after data collection that our study was affected by a surge in female participant signups, which led to high representation of female participants in our study. [↑](#footnote-ref-3)
4. We were notified by Prolific after data collection that our study was affected by a surge in female participant signups, which led to high representation of female participants in our study. [↑](#footnote-ref-4)
